# Bioapplications of Bacterial Cellulose Polymers Conjugated with Resveratrol for Epithelial Defect Regeneration

**DOI:** 10.3390/polym11061048

**Published:** 2019-06-15

**Authors:** En Meng, Chin-Li Chen, Chuan-Chieh Liu, Cheng-Che Liu, Shu-Jen Chang, Juin-Hong Cherng, Hsiao-Hsien Wang, Sheng-Tang Wu

**Affiliations:** 1 Division of Urology, Department of Surgery, Tri-Service General Hospital, National Defense Medical Center, Taipei 112, Taiwan; en.meng@gmail.com (E.M.); j0921713355@yahoo.com.tw (C.-L.C.); 2 Division of Cardiology, Department of Internal Medicine, Cardinal Tien Hospital, New Taipei City 231, Taiwan; chuanchiehliu@gmail.com; 3 School of Medicine, Fu-Jen Catholic University, New Taipei City 242, Taiwan; 4 Department of Physiology and Biophysics; Graduate Institute of Physiology, National Defense Medical Center, Taipei 114, Taiwan; chencheliu2002@gmail.com; 5 Division of Rheumatology/Immunology/Allergy, Department of Internal Medicine, Tri-Service General Hospital, National Defense Medical Center, Taipei 114, Taiwan; belle661011@gmail.com; 6 Department and Graduate Institute of Biology and Anatomy, National Defense Medical Center, Taipei 114, Taiwan; 7 Department of Gerontological Health Care, National Taipei University of Nursing and Health Sciences, Taipei 112, Taiwan; 8 Section of Urology, Cheng-Hsin Rehabilitation Medical Center, Taipei 112, Taiwan

**Keywords:** bacterial cellulose, biodegradable polymer biomaterials, epidermal reconstruction, tissue engineering scaffolds, wound healing

## Abstract

Excellent wound dressing is essential for effective wound repair and regeneration. However, natural polymeric skin substitutes often lack mechanical strength and hydrophilicity. One way to overcome this limitation is to use biodegradable polymers with high mechanical strength and low skin-irritation induction in wet environments. Bacterial cellulose (BC) is an attractive polymer for medical applications; unlike synthetic polymers, it is biodegradable and renewable and has a strong affinity for materials containing hydroxyl groups. Therefore, we conjugated it with resveratrol (RSV), which has a 4′-hydroxyl group and exhibits good biocompatibility and no cytotoxicity. We synthesized BC scaffolds with immobilized RSV and characterized the resulting BC/RSV scaffold with scanning electron microscopy and Fourier-transform infrared spectroscopy. We found that RSV was released from the BC in vitro after ~10 min, and immunofluorescence staining showed that BC was highly biocompatible and regenerated epithelia. Additionally, Masson’s trichrome staining showed that the scaffolds preserved the normal collagen-bundling pattern and induced re-epithelialization in defective rat epidermis. These results indicated that RSV-conjugated BC created a biocompatible environment for stem cell attachment and growth and promoted epithelial regeneration during wound healing.

## 1. Introduction

Successful wound treatment depends on competent care and continuous monitoring during the healing process. Deeper, wider, and chronic wounds require a higher level of clinical care. In the management of wound healing, the medical team collaborates to monitor and treat serious wounds; however, this approach entails considerable expenditures of time and money. In wound healing, immune cells are attracted to injury sites that are revascularized by hemostasis, and the connective-tissue matrix is produced by fibroblasts and keratinocytes to eventually induce re-epithelialization. This complex process is stimulated and regulated by various growth factors and cytokines. A previous study indicated that, compared with dry wound-treatment environments [1], wet conditions accelerate re-epithelialization and reduce pain and scar formation.

Cellulose, the most abundant known carbohydrate polymer, is naturally occurring, ubiquitous, and inexpensive. Bacterial cellulose (BC) is a biocompatible extracellular polysaccharide produced and secreted by the aerobic Gram-negative bacterium *Acetobacter xylinum* [2]. Previous studies of BC focused on its structure and physicochemical properties. An important property of BC is its chemical purity. BC is an unbranched polymer of β-1,4-linked glucopyranose residues [3,4,5]. The polymer chains of BC aggregate to form ~1.5-nm wide subfibrils as some of the thinnest naturally occurring fibers, with only sub-elemental cellulose fibers in the cambium of certain plants having comparable thickness. BC subfibrils crystallize into microfibrils and then into bundles and ribbons, which are typically 3 nm to 4 nm thick and 70 nm to 130 nm wide. Microbial cellulose ribbons are 1 nm to 9 nm long and form a dense reticulated structure stabilized by extensive hydrogen bonding. These unique structural properties enable BC use in paper, textile, and food industries and as a biomaterial in cosmetics and medicine. However, the range of its applications is largely dependent on cost and production scale. Therefore, basic studies are focused mainly on bacterial strains and improvements in the production process. BC has been investigated by several research groups as a scaffold for cartilage [6,7], wound dressing [8], dental implants [9,10], nerve regeneration [11], vascular grafts [12], and as a temporary skin substitute [13]. BC has excellent characteristics and a pore size (0.2 μm) appropriate for numerous applications. Nevertheless, it has inadequate bacterial and radiation resistance. Moreover, BC per se has no antimicrobial activity and does not prevent wound infection. Previous studies have attempted to confer antimicrobial efficacy upon BC by immersing it in silver nitrate solution or reducing it with zinc [14]; however, the products derived from these treatments exhibit low biocompatibility and are harmful to the environment. BC in wound dressings shows good cytocompatibility and histocompatibility [15] and provides a constantly moist environment conducive to skin regeneration. Additionally, BC enhances exudate absorption, which in turn increases fibrinous clotting and the uptake of necrotic tissue. Furthermore, the porous cellulose structure mimics the extracellular matrix (ECM) of skin and promotes tissue regeneration [15,16] as a result of the presence of highly porous, biocompatible, and biodegradable architecture. Unfortunately, these properties alone are insufficient to enable BC to serve as a wound dressing or skin-tissue substitute, as wound dressing must also simulate the ECM of the wound bed, accelerate wound healing, and reduce scar formation. In this study, we focused on the ability of BC applications for skin-regenerative medicine and wound healing by evaluating its efficacy in an animal model.

Type I collagen (COL) is the most abundant ECM component in skin. COL is responsible for the tensile strength of connective tissue and enables it to be stretched extensively without breaking [17]. Highly porous COL producing substantial quantities of ECM stimulate regeneration after severe injury of the skin [18,19], peripheral nerves [20], and conjunctiva [21]. Moreover, COL has replaced autografts during the treatment of skin loss in acutely burned patients [22], burn-scar revision [23], hand surgery [24], and skin wounds [25]. COL and BC are the most widely used biopolymers, because they target antibacterial, anti-inflammatory, proliferative, and remodeling processes and either directly interact with cells or are mediated by ECM during wound healing. Biopolymers modulate cellular behavior and trigger cell growth, differentiation, and the secretion of ECM components by fibroblasts and keratinocytes. Moreover, they are chemotactic for macrophages [15]. ECM proteins such as fibronectin activate immune cells such as macrophages, which remove neutrophils and debris from the wound site. Fibronectin induces monocytes to differentiate into macrophages, which in turn produce various cytokines and chemoattractants for fibroblasts and keratinocytes [26]. Furthermore, fibronectin activates the Wnt/β-catenin, the transforming growth factor (TGF)-β, the mitogen-activated protein kinase, and the phosphoinositide 3-kinase/Akt pathways in wound tissue [27]. However, ECMs alone have certain limitations in skin-wound repair. Their anti-inflammatory and antioxidant capacities are weaker than those of several drugs, and they can also exacerbate or prolong the inflammatory state and hinder wound healing and remodeling [28]. Inflammation is important in the elimination of infection and debris; however, protracted inflammation can cause tissue damage, increase scarring, and retard wound healing. Therefore, suitable regulation of the inflammatory response is necessary to ensure accelerated wound healing.

Resveratrol (RSV) has a 4′-hydroxyl group and exhibits good biocompatibility and no cytotoxicity. As a polyphenolic compound, RSV occurs naturally in red grape skins, red wine, peanuts, cranberries, and other fruits and reportedly exhibits low toxicity and is well tolerated in humans [29]. Additionally, RSV upregulates endothelial nitric oxide synthase (eNOs) and vascular endothelial growth factor (VEGF) and promotes angiogenesis in wound-healing experiments. Moreover, various RSV concentrations and longer treatment periods demonstrate efficacy on aged wounds [30]. We hypothesized that RSV could regulate inflammatory responses in wound healing. To test this hypothesis, we investigated the effects of BC and COL combined with RSV on epithelial regeneration and cutaneous wound healing. We evaluated the biocompatibility of various biopolymers with human adipose stem cells and the influences of these substances on epithelial differentiation. This pilot study revealed whether stem cells and epidermal tissue-engineering techniques regenerate epithelium in the urinary bladder and other organs. One of the major issues in tissue regeneration is the safety of cell therapy and polymer reconstruction; however, it is already recognized that relatively low health risks are associated with the aforementioned biopolymers and stem cell methods according to animal studies.

## 2. Materials and Methods

### 2.1. BC Fabrication

BC sheets were purchased from the Far Eastern Group (BF10005; The Far Eastern Group, Taipei, Taiwan, ROC). The BC was produced by *Acetobacter xylinum* cultured in Buffered Schamm and Hestrin’s broth for 2 d. The BC was transferred to coconut juice (pH 4.0–4.4; 30 °C) for 2 d then washed in 0.1 M NaOH (aq) at 90 °C to 95 °C to remove bacterial toxins. BC fibers were then bleached with 0.25% H_2_O_2_ at 45 °C for 30 min then washed in water, followed by compression into a sheet and dehydration with acetone until their water content was <15% and their thickness was 2 mm. BC was stored in an electric drying cabinet.

### 2.2. Preparation of BC Scaffolds Containing RSV

RSV (R5010; MW 228.2) was purchased from Sigma-Aldrich (St. Louis, MO, USA). A stock solution was prepared by dissolving 50 mg RSV in absolute ethanol and stirring at 200 rpm for 1 h. This solution was dispersed in 0.1 M phosphate-buffered saline (PBS; pH 6.8) and adjusted to a 50-μM working solution. RSV solution was applied to the BC scaffolds with a syringe, with a final RSV concentration on each BC scaffold of 1.43 μg cm^−2^. The RSV-doped BC scaffold (BC/RSV) was placed under a laminar flow hood, dried, and stored in the dark at 25 °C.

### 2.3. Preparation of a Type I COL Scaffold Containing RSV

Type I COL solution (C9791; 0.25% *w*/*v*; Sigma-Aldrich) was dissolved in 1% (*v*/*v*) acetic acid. An 8 μg mL^−l^ RSV solution was prepared by dissolving the stock RSV solution in 100% ethanol, followed by combining the COL and the RSV solutions with stirring for 24 h at 25 °C. COL/RSV aliquots of 0.275 mL were placed in glass vials and gradually cooled at a rate of 2 °C min^−l^ until the samples reached −20 °C. The frozen aliquots were then placed in a freeze-drying chamber and further cooled at a rate of 2 °C min^−l^ until they reached −45 °C. The samples were then processed in a freeze-dryer (FD24-4S; Kingming, Taipei, ROC) at −45 °C under a 300-mbar vacuum for 24 h, followed by the addition of 0.5 mL of 2.5% (*w*/*v*) PCL/dichloromethane solution to each frozen COL or COL/RSV scaffold and incubation for 30 min. The vial lids were then removed to allow solvent evaporation.

### 2.4. Scanning Electron Microscopy (SEM)

The surface morphology of the lyophilized scaffolds and the cells grown on them was examined with a Hitachi S–3000N SEM (Hitachi High Technologies, Krefeld, Germany). The samples were fastened to carbon stubs and mounted on aluminum stubs. SEM images were acquired under an accelerating voltage of 1.5 kV at a working distance of ~15.0 mm and at 500× to 1000× magnification.

### 2.5. Fourier-Transform Infrared (FT-IR) Spectroscopy

Attenuated total reflectance (ATR) FT-IR spectra of the samples were recorded on a Nicolet 8700 spectrometer (Thermo Fisher Scientific, Waltham, MA, USA) fitted with a high-performance diamond single-bounce ATR accessory (wavenumber range, 4000–400 cm^−1^; resolution, 1 cm^−1^; 16 scans/spectrum). Mercury-cadmium-telluride was used for infrared detection.

### 2.6. Efficiency of RSV Release from the Scaffold

BC/RSV or COL/RSV scaffolds were immersed in artificial saliva solution (Biotène; GlaxoSmithKline, London, UK) and shaken at ±37 °C. The medium was sampled at 0 min, 2 min, 4 min, 8 min, 16 min, 30 min, and 60 min, and RSV absorbances were measured at 350 nm by UV spectrophotometry (SYNERGY HTX; BioTek, Winooski, VT, USA). All assays were performed in triplicate.

### 2.7. In Vitro Biocompatibility

Human adipose stem cells line (hASCs) provided by Dr. Cherng were cultured in supplemented keratinocyte serum-free medium (Life Technologies Ltd., Paisley, Scotland, UK) with 10% fetal bovine serum (Hyclone, Logan, UT, USA) at 37  °C in humidified air containing 5% CO_2_. The scaffolds were sterilized by UV irradiation for 18 h, followed by hASC seeding onto the scaffolds at a density of 1 × 10^6^ cells cm^−2^ and incubating under 5% CO_2_ at 37 °C. The culture medium was replaced every 2 d, and the samples were observed by immunocytochemical staining. Experiments were performed in triplicate and repeated three times with similar results.

### 2.8. Animal Model of a Surgical Epidermal Defect

Eighteen male Sprague-Dawley rats (250–300 g) were purchased from Bio-LASCO Co. Ltd. (Taipei, Taiwan). Two animals were housed per cage in a pathogen-free facility. The experimental protocol was reviewed and approved by the Institutional Animal Care and Use Committee: IACUC-17-059 at the National Defense Medical Center. The rats were anaesthetized with intraperitoneal chloral hydrate (0.4 mg g^−1^). For the wound-healing experiment, rats were randomly separated into five groups: lesion control, BC, BC/RSV, COL, and COL/RSV. Two wounds 1 cm in diameter were created on the dorsum of each rat, and the experimental scaffolds were bound to the wounds with biocompatible mucilage. The control wounds were left untreated and covered with plain medical gauze. Medical gauze was also used to bind the dressings onto the wounds and replaced every 2 d as required. Rats were kept in individual cages, and on days 3, 7, and 14 after wound creation, the lesions were photographed, and the rats were euthanized by anesthesia overdose. All experiments were performed in duplicate and repeated at least three times.

### 2.9. Histologic Examination

The wounds and their surrounding skin tissues were excised and used for histologic evaluations. Samples collected on days 3, 7, and 14 were fixed in 10% formalin with sucrose, and the tissues were snap-frozen on dry ice in optimal cutting temperature embedding medium. The frozen tissues were sectioned into slices 30-µm thick and treated with hematoxylin and eosin and Masson’s trichrome (HT-1079+HT-15; Sigma-Aldrich) stains. The tissue sections were observed under a light microscope and photographed with a SPOT-RT digital camera (Diagnostic Instruments, Detroit, MI, USA). Re-epithelialization and COL deposition were the criteria used for histochemical examinations. All staining was performed in triplicate and repeated three times with similar results.

### 2.10. Immunofluorescence

Both hASC seeding in the scaffolds and the skin-wound sites were subjected to immunocytochemical/histochemical examinations. The samples were fixed with 4% paraformaldehyde and cut into slices 30-µm thick by cryosection. The samples were treated with 0.2% Triton X-100 for 30 min, followed by three washes with PBS for 5 min/wash. Nonspecific binding sites were blocked with 10% normal goat serum (Vector Laboratories Ltd., Burlingame, CA, USA), and the samples were incubated with the primary antibodies anti-fibronectin (1:500, monoclonal rabbit), anti-CK-14 (1:500, monoclonal mouse), anti-involucrin (1:500, monoclonal rabbit), anti-octomer-binding protein 4 (OCT4; 1:500, polyclonal rabbit), and anti-nestin (1:500, monoclonal mouse) (all from Santa Cruz Laboratories, Dallas, TX, USA) and incubated for 2 h at room temperature. The samples were washed three times with PBS for 5 min/wash, followed by incubation with the secondary antibodies fluorescein isothiocyanate-conjugated anti-rabbit (1:1000; Jackson ImmunoResearch, West Grove, PA, USA) and rhodamine-conjugated anti-mouse (1:1000; AnaSpec, Fremont, CA, USA) at room temperature for 1 h. To visualize nuclei, sections were counterstained with Hoechst 33342 (1:5000; AnaSpec) for 15 min. Fluorescent images were obtained with an inverted fluorescent microscope (Axio Lab.A1; Carl Zeiss AG, Oberkochen, Germany) fitted with a camera (Zeiss AxioCam ICm1; Carl Zeiss AG). All staining was performed in triplicate and repeated three times with similar results.

### 2.11. Wound-Area Measurements and Statistical Analysis

Wound area was documented with a digital camera (Nikon Coolpix 4100; Nikon, Tokyo, Japan) on days 3, 7, and 14, and images were analyzed using ImageJ software (National Institutes of Health, Bethesda, MD, USA) by calculating the wound margin area and pixel area. Data are expressed as the mean ± standard deviation of the respective replicates (*n* = 3 or 4). Statistical differences between treatment means were determined with Student’s *t* test and one-way analysis of variance. All statistical analyses were performed in Microsoft Excel 2016 (Microsoft Corp., Redmond, WA, USA), and a *P* < 0.05 was considered statistically significant.

## 3. Results and Discussion

### 3.1. FT-IR

**Figure 1:**
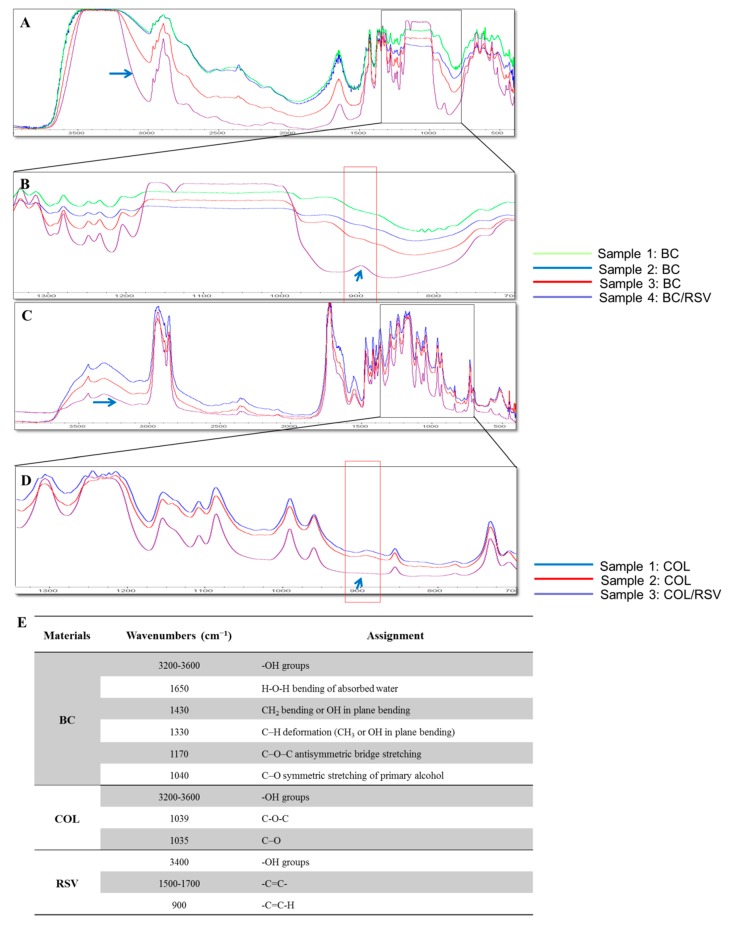
FT-IR spectrum of the resveratrol (RSV) particle. (**A**) Bacterial cellulose (BC)/RSV scaffold (purple line), (**B**) enlargement of (**A**), (**C**) type I collagen (COL)/RSV scaffold, and (**D**) enlargement of (**C**). Black squares are enlarged areas, and red squares indicate the region of the RSV spectrum. (**E**) FT-IR identification of the RSV regions in the scaffolds.

The molecular interactions of the BC and the COL scaffolds (Figure 1) containing RSV were qualitatively investigated by FT-IR spectroscopy. The spectra for the BC and the BC/RSV scaffolds (Figure 1A) revealed strong bands for structures, such as H–O–H near 1490 cm^−1^ and the C–H deformation (CH_3_ or OH in-plane bending) at 1330 cm^−1^. Bands at 1170 cm^−1^ and 1040 cm^−1^ corresponded to antisymmetric C–O–C bridge stretching and symmetric C–O stretching, respectively. These results corroborated previous studies indicating that *A. xylinum* produces cellulose with an IR spectrum in the region of 3400 cm^−1^ to 3200 cm^−1^, which indicates a relative abundance of cellulose Iα [31,32,33]. The RSV spectrum in the BC/RSV scaffold (Figure 1B) was characterized by C–C ring stretching at 1300 cm^−1^, a –C=C–H band corresponding to *trans* olefinic bands at 900 cm^−1^, and C–C aromatic double-bond stretching at ~1500 cm^−1^ to 1700 cm^−1^ [34]. The COL scaffold (Figure 1C) presented absorption peaks at 1035 cm^−1^ and 1039 cm^−1^, which were associated with C–O stretching vibrations and C–O–C stretching, respectively [35]. The C–C aromatic double bond and the olefinic stretching showed peaks overlapping at 1000 cm^−1^ to 1600 cm^−1^ (Figure 1C). These were attributed to the absorption of CH_2_, CH_3_, C–N, and N–H moieties of COL [35]. Strong absorptions in the region of 3000 cm^−1^ to 2800 cm^−1^ and 1800 cm^−1^ to 1600 cm^−1^ were assigned to amides A and B and amides I and II of COL, respectively [36,37]. This region corresponded to the alkene carbon–hydrogen bond. The regions of the structures are listed in Figure 1E. The 900 cm^−1^ peak was absent from the COL/RSV scaffold (Figure 1D) as compared with the BC/RSV group; however, the COL/RSV scaffold was a freeze-dried biopolymer. Therefore, it remains to be determined whether the RSV functional group was altered after the freeze-drying process.

### 3.2. SEM

**Figure 2:**
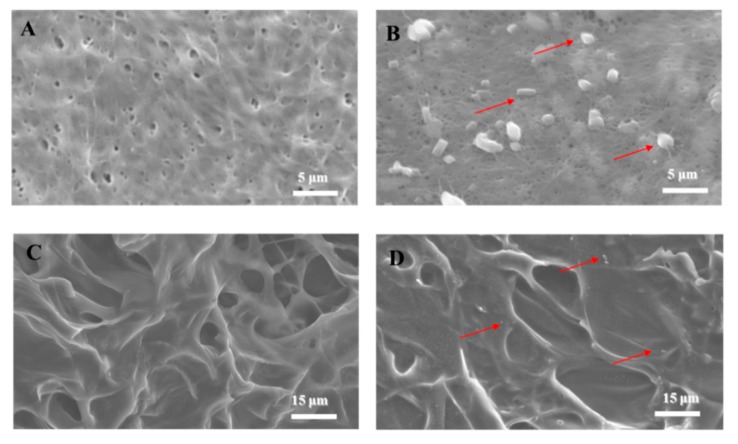
SEM analysis. (**A**) BC scaffold, (**B**) BC/RSV scaffold, (**C**) COL scaffold, and (**D**) COL/RSV scaffold. Red arrows indicate RSV particles. Scale bars = 5 μm (**A**,**B**) and 15 μm (**C**,**D**).

In tissue engineering, SEM analysis discloses the morphological structure of polymers in biomaterials (Figure 2). The BC scaffold (Figure 2A) comprised fibers with a pore diameter of ~0.2 μm, and the surface of the COL scaffold (Figure 2C) had pores with a diameter of ~8 μm. The RSV spread evenly on the biomaterial surfaces after application (Figure 2B,D; red arrows), with a size range of 0.1 µm to 2 μm. The COL/RSV scaffold (Figure 2D) was larger than the BC/RSV scaffold, with the former having relatively looser holes and small- to medium-sized pores with undefined shapes. The SEM image showed fewer RSV particles in the COL scaffold relative to the BC scaffold. This discrepancy might account for the apparent absence of the –C=C–H band observed by FT-IR (Figure 1D).

**Figure 3:**
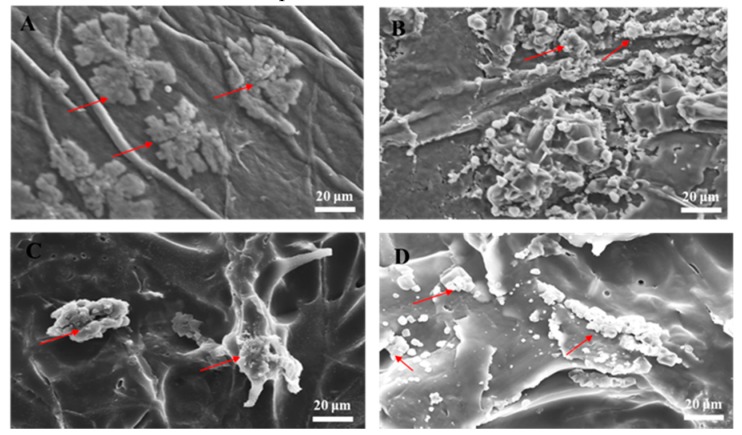
SEM images of human adipose stem cells line (hASCs) grown on biomaterials for 7 d. (**A**) BC scaffold, (**B**) BC/RSV scaffold, (**C**) COL scaffold, and (**D**) COL/RSV scaffold. Red arrows indicate hASCs. Scale bar = 20 μm.

In tissue engineering, scaffolds serve as temporary platforms for tissue formation and promote cellular migration, new ECM formation, tissue ingrowth, and nutrient and metabolic waste transport [38]. Therefore, the scaffold must be biocompatible (nontoxic) and provide an environment conducive to cell adhesion and function. We incubated hASCs on the biomaterials for 7 d and inspected them by SEM. Our analysis revealed that the cells adhered and grew well on the biomaterials, suggesting their biocompatibility for ex vivo stem cell attachment. We then compared the SEM images of hASCs seeded on the BC (Figure 3A), the BC/RSV (Figure 3B), the COL (Figure 3C), and the COL/RSV (Figure 3D) scaffolds. All tested biomaterials were highly biocompatible with hASCs, and their surfaces fostered cell expansion.

### 3.3. Measurement of RSV Release over Time

**Figure 4:**
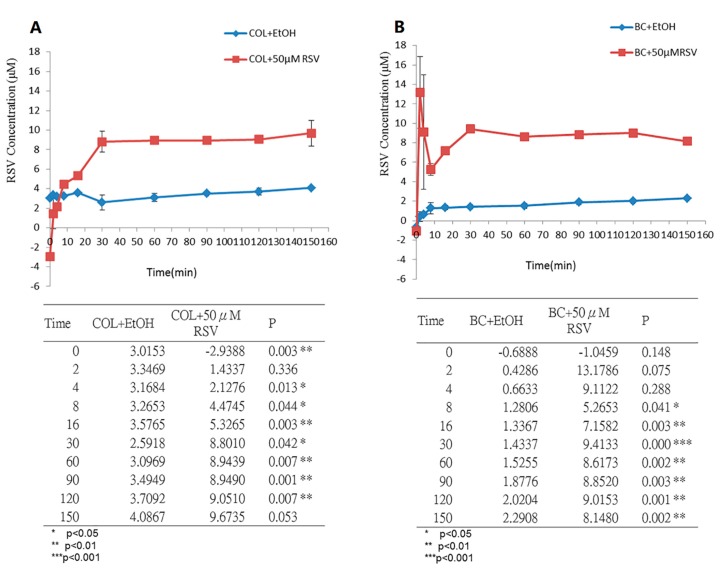
Monitoring RSV release from biomaterials in artificial saliva solution at 37 °C. (**A**) BC/RSV scaffold and (**B**) COL/RSV scaffold.

In clinical applications, the release time of natural pharmacologically active biomaterials from human tissues needs to be evaluated in order to assess their efficacy. We incubated the BC/RSV and the COL/RSV scaffolds with artificial saliva solutions at 37 °C to simulate human body fluids. Acute inflammation responses, including fluid and plasma exudation into tissue and neutrophil and macrophage accumulation, commonly occur at the start of the healing process. RSV is a potent anti-inflammatory modulator [39]; therefore, it must be released in a timely manner to ensure its inhibition of severe inflammation. Equilibria of the release of RSV from the BC/RSV and the COL/RSV scaffolds were achieved after 60 min (Figure 4A) and 30 min (Figure 4B), respectively, with RSV concentrations released from the BC/RSV and the COL/RSV scaffolds of 8.6 µM (*P* < 0.05) and 8.8 µM (*P* < 0.01), respectively. Our results suggested that RSV-treated COL was more appropriate for medical applications as compared with RSV-treated BC, because the former releases RSV faster and at a higher concentration than the latter. Because BC harbors multiple hydroxyl groups, it exhibits a strong affinity for RSV, thereby precluding its rapid release [40].

### 3.4. Functional Biocompatibility In Vitro

**Figure 5:**
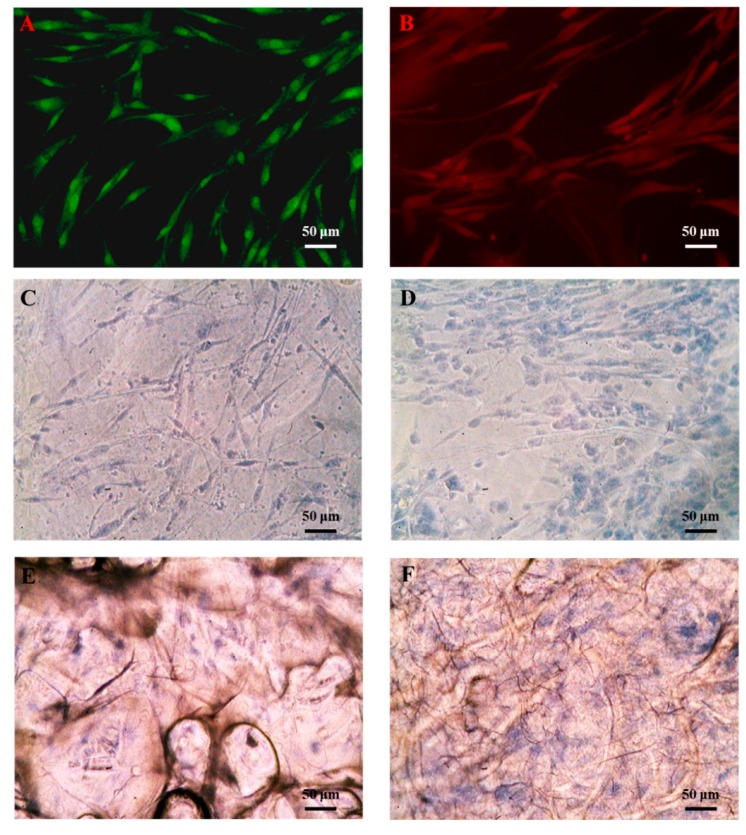
Immunofluorescence staining of stem cells in the presence of hASCs. (**A**) Octamer-binding transcription factor 4 (OCT-4) and (**B**) bone morphogenic protein (BMP)4. Trypan blue staining of hASCs grown on biomaterials for 7 d. (**C**) BC, (**D**) BC/RSV, (**E**) COL, and (**F**) COL/RSV scaffolds. Scale bar = 50 μm.

Trypan blue staining was used to observe the cells in the biomaterials and confirm scaffold biocompatibility with the cells (Figure 5). Compared with the BC and the COL scaffolds (Figure 5C,E), stronger positive Trypan blue staining was detected in the BC/RSV (Figure 5D) and the COL/RSV (Figure 5F) scaffolds, suggesting that the latter two harbored relatively more living cells. The addition of RSV to BC or COL apparently enhanced cell growth, which agreed with previous studies [41,42,43]. This observation might be explained by RSV promoting cellular self-renewal by inhibiting apoptosis and senescence. In the present study, we used octamer-binding transcription factor 4 (OCT-4) and bone morphogenic protein (BMP)4-positive staining of hASCs to confirm stem cells by immunofluorescence staining (Figure 5A,B).

### 3.5. Immunostaining

**Figure 6:**
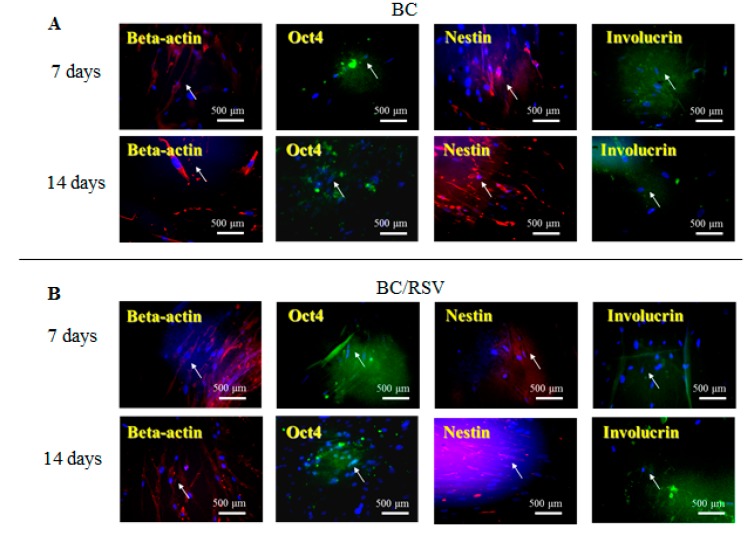
Representative images showing BC and BC/RSV scaffold biocompatibility with hASCs. (**A**) BC and (**B**) BC/RSV scaffolds. Images are of formalin-fixed, frozen, and immunostained stem cells for OCT4, nestin, and the keratinocyte-differentiation marker involucrin. β-actin was used as an internal staining control. White arrows indicate positive antibody expression in the scaffold. Nuclei were counterstained with Hoechst 33342 (blue). Scale bar = 500 μm.

**Figure 7:**
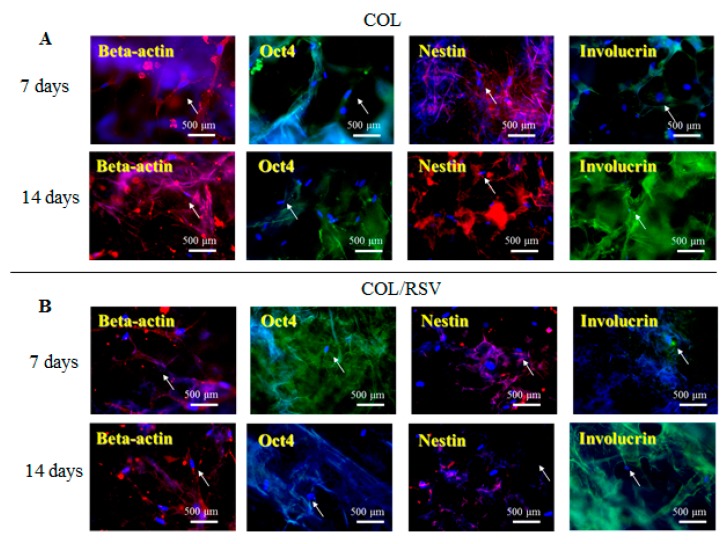
Representative images showing COL and COL/RSV biocompatibility with hASCs. (**A**) COL and (**B**) COL/RSV scaffolds immunostained with the stem cell biomarkers OCT4, nestin, and the keratinocyte-differentiation marker involucrin. β-actin was used as an internal staining control. White arrows indicate positive antibody expression in the scaffold. Nuclei were counterstained with Hoechst 33342 (blue). Scale bar = 500 μm.

An in vitro study was conducted using the BC and the COL scaffolds with and without RSV, and stem cell and keratinocyte differentiation biomarkers β-actin, OCT4, nestin, and involucrin were used to visualize activation of cells implanted into the BC, the COL, the BC/RSV, and the BC/COL scaffolds, respectively. β-actin is a cytoskeleton marker that indicates the position and the shape of the stem cells on the scaffolds [44]. OCT4 and nestin are stem cell markers, with OCT4 expressed in the early stages of stem cell development and again in the later stages [45]. Involucrin is expressed in the late stages of stem cell development during terminal diffraction and also represents a keratinocyte-precursor marker [46]. Relative to the BC and the COL scaffolds alone (Figure 6A and Figure 7A), we observed an increased positive staining of stem cell markers on the BC/RSV (Figure 6B) and the COL/RSV (Figure 7B) scaffolds after a 14-d incubation, suggesting both more stem cells and more mature cells on the RSV-treated scaffolds than on the untreated scaffolds. A previous study reported that RSV facilitates mesenchymal stem cell viability, osteogenesis, and paracrine secretion in vitro [47]. Another study indicated that RSV used in stem cell-aggregate engineering generated stem cells attached to newly formed ECM [48]. These results indicate that the RSV on the BC and the COL scaffolds provided a high level of biocompatibility and a suitable microenvironment for cell growth. Several studies reported that RSV might regulate stem cell survival, self-renewal, and differentiation and prevent stem cell damage, carcinogenesis, and aging while also promoting the regeneration of other types of tissue [48,49,50]. For example, RSV might support regeneration and reconstruction of the urinary bladder [51].

### 3.6. Skin Reconstruction in An Animal Epidermal-Defect Model

**Figure 8:**
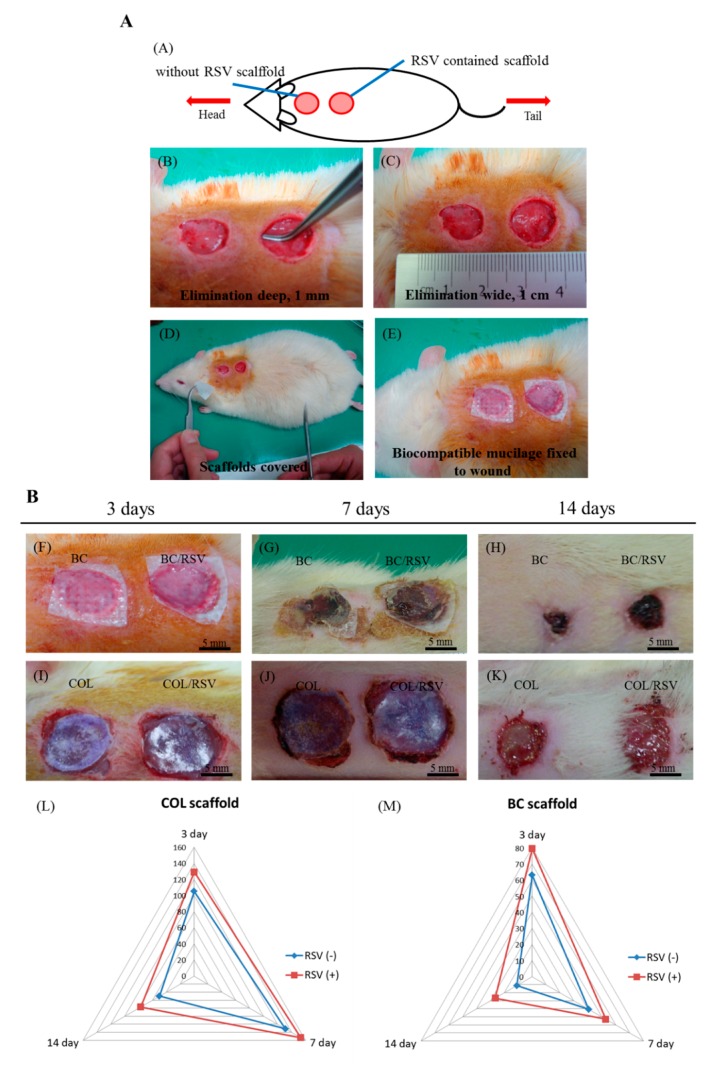
Effect of scaffolds on dermal wound healing. (**A**) Skin defects induced on the back of a rat and filled with scaffolds; (**B**) wound contraction observed in each scaffold group on days 3, 7, and 14 after injury. [(**L**),(**M**)] Semi-quantitatively measure the wound area of the epidermal defect in each material group.

Tissue homeostasis and the protective function of the skin are restored by wound healing, which involves several complex cellular and biochemical processes. Dermal collagen remodeling and scar budding are important tissue-repair processes during the maturation phase [52]. Therefore, we examined the influences of the BC/RSV and the COL/RSV scaffolds on a rat epidermal-defect model (Figure 8A–E). The BC/RSV and the COL/RSV scaffolds were embedded in the wound sites, and the BC and the COL scaffolds were used as controls. Representative images of wound healing associated with different treatments and at various time intervals are shown in Figure 8F–K. After 14 d, the wound site was smallest in the BC scaffold and the surrounding normal skin shrank (Figure 8H), whereas the tissue around the wound site was smooth in the COL and the COL/RSV scaffolds, and wound closure was delayed (Figure 8K). Quantitative analysis of the wound area (mm^2^) in each material group is shown in the radar chart (Figure 8L,M), which shows a trend of wound healing. The results indicated that, regardless of RSV incorporation in the BC or the COL scaffolds, we observed maximal decreases in wound area at 14 d after dressing. Masson’s trichrome staining used to highlight COL fiber formation and epidermal histology during the healing process revealed that, although the RSV-containing scaffolds did not display excellent performance in reducing wound area, the wound-healing capacities of these materials were confirmed.

**Figure 9:**
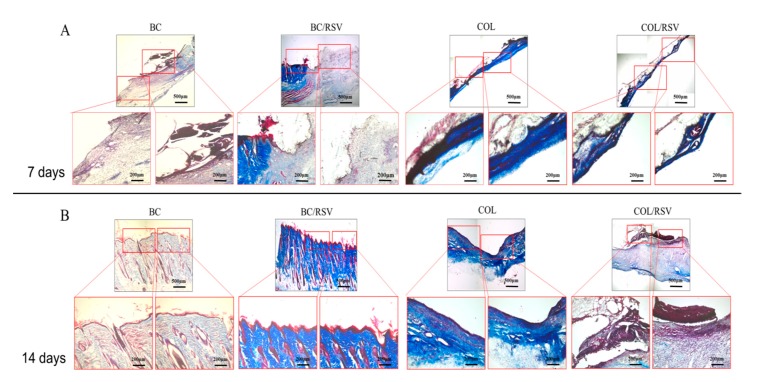
Histological assessment of scaffold implants in skin defects after 7 and 14 d. (**A**) Masson’s trichrome staining demonstrated that COL bundles formed after 7 d in the wound treated with RSV-loaded BC and COL scaffolds; (**B**) after 14 d, remodeling progress was improved in the presence of the BC/RSV scaffolds relative to the COL/RSV scaffolds. Relatively greater COL accumulation and deposition were observed in the COL scaffolds. Light micrographs of Masson’s trichrome staining show that the BC/RSV scaffolds preserved normal COL-bundling patterns and orientation.

To further analyze whether RSV improves wound healing, we evaluated the level and the quality of epithelial regeneration in wound tissue. During wound healing, fibroblasts and myofibroblasts modulate cellular functions, maintain cellular-tensional homeostasis, and shrink and reorganize the COL matrix during tissue development and repair [53]. Wound contraction is affected by COL synthesis and compaction of granular tissue. Masson’s trichrome staining revealed that the BC/RSV scaffolds induced COL synthesis in skin tissues over time (Figure 9A,B). A dense blue color appeared in the BC/RSV-treatment area after 14 d (Figure 9B), and there was a uniform distribution of the stratum spinosum, which integrated with the granular tissue in the epidermis. RSV has an affinity for estrogen receptor (ER)α and ERβ found on keratinocytes, fibroblasts, and macrophages and stimulates the production of COL types I and II and ECM [54,55]; however, progressive COL production might also induce scar formation (Figure 8H) [56]. The COL scaffold promoted early stratum-corneum-barrier regeneration and synthesis of a COL matrix in the wound; however, granular tissue did not form until 14 d after the injury. Histologic analysis confirmed that RSV on the COL scaffold inhibited COL synthesis in the skin tissue over time (Figure 9A,B). Indeed, loose COL-fiber staining was detected after 14 d (Figure 9B), with histological images suggesting that the epidermal regeneration was suboptimal. Therefore, we evaluated re-epithelialization and wound-tissue regeneration via immunofluorescence staining in order to assess the potential of RSV and the scaffolds to promote wound-healing regeneration. Staining results confirmed the mechanism by which RSV-incorporated BC or COL scaffolds affected wound healing.

### 3.7. Re-Epithelialization of the Epidermal Debride Area in Skin Reconstitution

**Figure 10:**
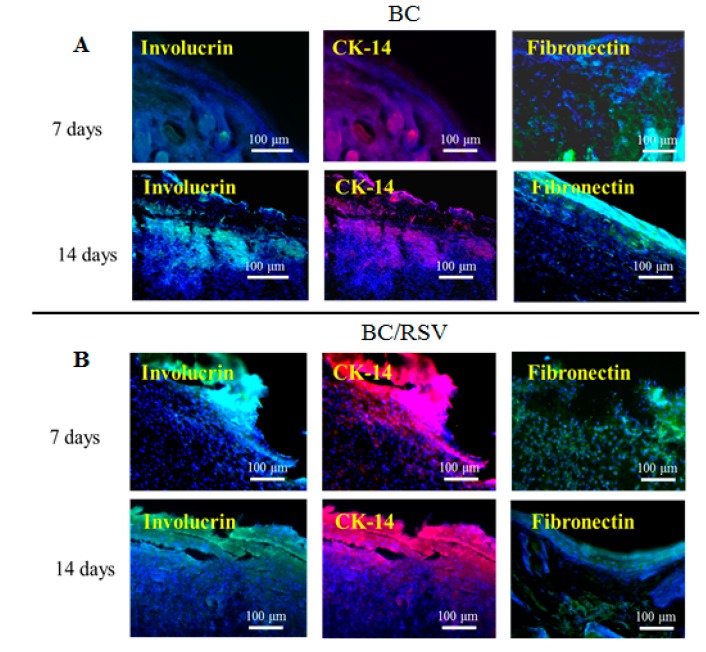
Keratinocyte differentiation on BC- and BC/RSV-scaffold implants in skin defects after 7 and 14 d. Implant areas were detected by immunofluorescence staining for involucrin (green) and CK-14 (red) in wound tissue for different groups at 7 d after implantation. The BC/RSV group showed relatively higher levels of involucrin- and CK-14-positive keratinocytes. The re-epithelialization marker fibronectin (green) was present in the BC-scaffold group at 14 d after implantation. Nuclei were counterstained with Hoechst 33342 (blue). Scale bar = 100 μm.

Re-epithelialization is a vital part of wound closure. Fibroblasts are recruited to rebuild the dermal layer while keratinocytes migrate from the edges of the wound and re-epithelialize the provisional matrix [45]. Immunohistochemical analysis indicated the presence of markers involucrin and CK-14 in the regenerative area, suggesting keratinocyte differentiation [57], and the presence of fibronectin suggested dermal-fibroblast cell behavior during wound repair [27]. Immunostaining (Figure 10A,B) revealed that the BC scaffold expressed involucrin, which is indicative of keratinocyte renewal, although expression was delayed until 14 d after induction of the epidermal defect. CK-14, a marker of mature keratinocytes, was expressed in the BC/RSV group, and the fibroblast marker fibronectin was expressed in the BC and the BC/RSV scaffolds at 14 d after implantation. Therefore, these scaffolds promoted both epidermal tissue regeneration and re-epithelialization during wound repair. Additionally, the BC/RSV scaffold (Figure 10B) upregulated the epithelial biomarkers to a greater degree than the BC scaffold alone. This finding suggested that the combination of BC and RSV attracted fibroblasts and keratinocytes to the wound site and induced their differentiation. RSV might enhance re-epithelialization by activating sirtuin-1 (SIRT1) [58], which plays vital roles in wound repair by regulating epidermal re-epithelialization, dermal granular-tissue formation, keratinocyte migration, cytokine expression, TGF-β signaling, and oxidative stress [59]. The functionality and the long-term performance of native tissue depend on its successful integration with biomaterials or implants. One motivation for the present study was the ability of BC to be absorbed and integrated into various living tissues [60]. In the present study, we found that the BC scaffold was biocompatible and did not induce immune reactions, chronic inflammatory responses, or neurotoxicity.

**Figure 11:**
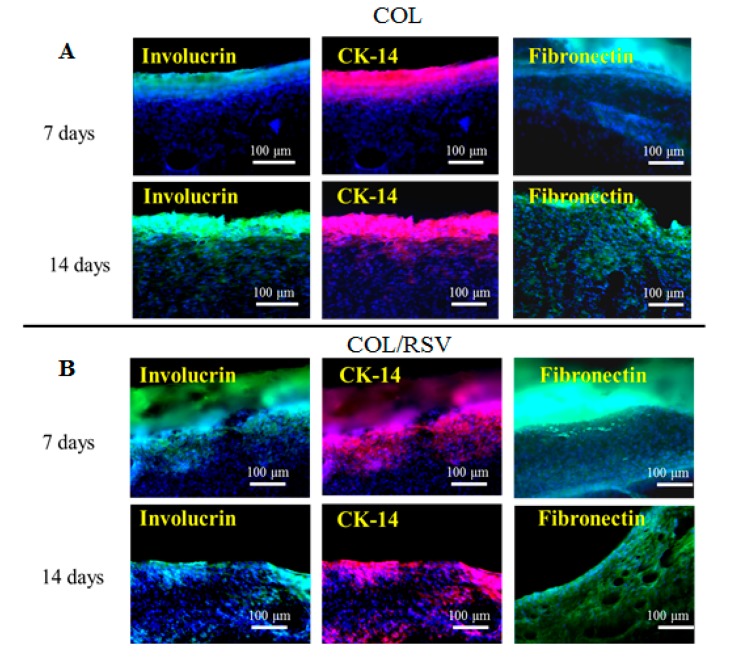
Keratinocyte differentiation on COL- and COL/RSV-scaffold implants in skin defects after 7 and 14 d. The COL/RSV group showed relatively higher levels of involucrin- (green) and CK-14- (red) positive keratinocytes. Nuclei were counterstained with Hoechst 33342 (blue). Scale bar = 100 μm. The COL and the COL/RSV scaffolds displayed fibronectin (green) expression, indicating ECM formation and re-epithelialization during wound healing.

Involucrin, CK-14, and fibronectin were upregulated on the COL scaffold, although their levels did not differ from those on the COL/RSV scaffold at 14 d after treatment (Figure 11A,B). COL-based material accelerates wound closure by improving the quality of healing, increasing COL deposition and maturation [61], and enhancing macrophage recruitment to the wound site. In the present study, we found that the COL/RSV scaffold (Figure 11B) did not induce re-epithelialization during wound healing any more effectively than the BC/RSV scaffold. Inclusion of RSV with either COL or BC was used in subsequent wound-healing experiments, because each displayed a different effect on epithelial cells in treated wounds.

## 4. Conclusions

Here, we described the development of stable, composite, RSV-based polymers for use as potential wound dressings to promote healing. Our data revealed that both BC and COL scaffolds containing RSV displayed excellent biocompatibility. RSV-based tissue engineering might effectively improve functionality in epidermal defects and facilitate their regeneration. BC and COL polymers differ in terms of their optimal functional properties as wound dressings. One limitation of the present study is the need to clarify whether the freeze-drying procedure changes the functional groups in the RSV-containing scaffolds. Our findings might enhance the clinical application of various regenerative treatments. It is necessary to elucidate the progression and the development of cellular differentiation in the stratified epithelial layer during tissue regeneration. Our future research will investigate highly plastic biological materials co-cultured with autologous stem cells for use in therapeutic urinary tract regeneration. Moreover, our quantitative validation of the infectious, the toxicological, and the immunological safety of the BS/RSV and the COL/RSV scaffolds promotes their use in human clinical trials. These procedures could promote the use of such scaffolds as surgical dressings and carriers for cell therapy, as well as for other clinical applications.
